# Incidence and risk of advanced age-related macular degeneration in eyes with drusenoid pigment epithelial detachment

**DOI:** 10.1038/s41598-022-08626-x

**Published:** 2022-03-18

**Authors:** Taiyo Shijo, Yoichi Sakurada, Koji Tanaka, Akiko Miki, Atsushi Sugiyama, Hajime Onoe, Aya Chubachi, Wataru Kikushima, Yu Wakatsuki, Seigo Yoneyama, Ryusaburo Mori, Kenji Kashiwagi

**Affiliations:** 1 Department of Ophthalmology, Faculty of Medicine, University of Yamanashi, Shimokato 1110, Chuo, 409-3821 Yamanashi Japan; 2 Department of Ophthalmology, Nihon University School of Medicine, Tokyo, Japan; 3 Department of Surgery, Division of Ophthalmology, Kobe University Graduate School of Medicine, Kobe, Japan

**Keywords:** Diseases, Medical research, Risk factors

## Abstract

To investigate the incidence and risk of advanced age-related macular degeneration (AMD), including geographic atrophy (GA) and macular neovascularization (MNV), in eyes with drusenoid pigment epithelial detachment (PED). Eighty-five eyes with drusenoid PED from 85 patients (77.2 ± 7.0 years, male/female: 44/41) were included in this study. Patients were followed up every 1–3 months via spectral-domain optical coherence tomography (SD-OCT) and color fundus photography. If exudation was observed on SD-OCT, fluorescein and indocyanine green angiography were performed to confirm the MNV subtype accordingly. The maximum follow-up period was 60 months. During the study period, GA developed in 8 eyes while MNV also developed in 8 eyes. The Kaplan–Meier estimator revealed that the cumulative incidence for 60 months was 17.9% and 12.2% for GA and MNV, respectively. In eyes developing MNV, retinal angiomatous proliferation was the most common. Cox regression analysis revealed that baseline PED width was the only factor associated with advanced AMD. (*p* = 0.0026, Cox regression analysis). The 5-year cumulative incidence of advanced AMD, including GA and MNV, was approximately 30% in eyes with drusenoid PED among the Japanese elderly. A larger baseline PED width was the only risk factor for advanced AMD.

## Introduction

Macular drusen are the hallmark lesions of age-related macular degeneration (AMD) along with retinal pigment abnormalities^1^. Drusenoid pigment epithelial detachment (PED) is characterized by the elevation of the retinal pigment epithelium (RPE) apart from Bruch’s membrane and aggregation/coalescence of macular drusen, although its pathogenesis has not been fully understood^2,3^. It is considered to be a clinical spectrum of non-exudative AMD.

The Age-related Eye Disease Study (AREDS) defined a drusenoid PED as round confluent drusen larger than 350 µm on color fundus photography .This study longitudinally investigated the natural history of eyes with drusenoid PEDs, reporting that advanced AMD developed in 42% of eyes, including 19% of eyes with geographic atrophy (GA) and 23% eyes with macular neovascularization (MNV) within 5 years^4^. Drusenoid PEDs are considered a strong risk factor for progression to advanced AMD.

A recent study using spectral-domain optical coherence tomography (SD-OCT) revealed that drusenoid PED volume slowly increases during the biogenesis stage and rapidly collapses with changes in RPE, including RPE thickening and migration, resulting in GA^5^. However, there have been few reports investigating the characteristics of MNV secondary to drusenoid PEDs and according to previous reports, drusenoid PEDs are exclusively seen in Caucasians.

In the present study, we investigated the incidence and risk factors of advanced AMD in Japanese elderly with drusenoid PED using SD-OCT and fluorescein/indocyanine green angiography.

## Results

A total of 85 eyes from 85 patients with drusenoid PED were included in this study. Table 1 shows the baseline demographic and clinical characteristics of patients with drusenoid PED. Of 51 patients with MNV in the contralateral eye, 23 eyes, 10 eyes, 12 eyes, and 6 eyes showed neovascular AMD, polypoidal choroidal vasculopathy, retinal angiomatous proliferation (RAP), and scarring, respectively. There were no patients involving GA in the contralateral eye.

**Table 1:** Baseline demographic characteristics of patients with drusenoid pigment epithelial detachment.

	DPED (n = 85)
Mean age (years)	77.2 ± 7.0
Gender Male	44 (51.8%)
Presence of RPD	19 (22.4%)
Presence of MNV in the contralateral eye (Category 4 in AREDS)	51 (60%)
SCT (µm)	214.4 ± 74.4
DPED height (µm)	206.1 ± 127.1
DPED width (µm)	1652.2 ± 1072.2
Follow up period(months)	33.2 ± 21.6
Baseline log MAR BCVA	0.09 ± 0.29

RPD: reticular pseudodrusen, MNV: macular neovascularization, SCT: subfoveal choroidal thickness, DPED: drusenoid pigment epithelial detachment, log MAR: logarithm minimum angle of resolution, BCVA: best-corrected visual acuity.

**Figure 1:**
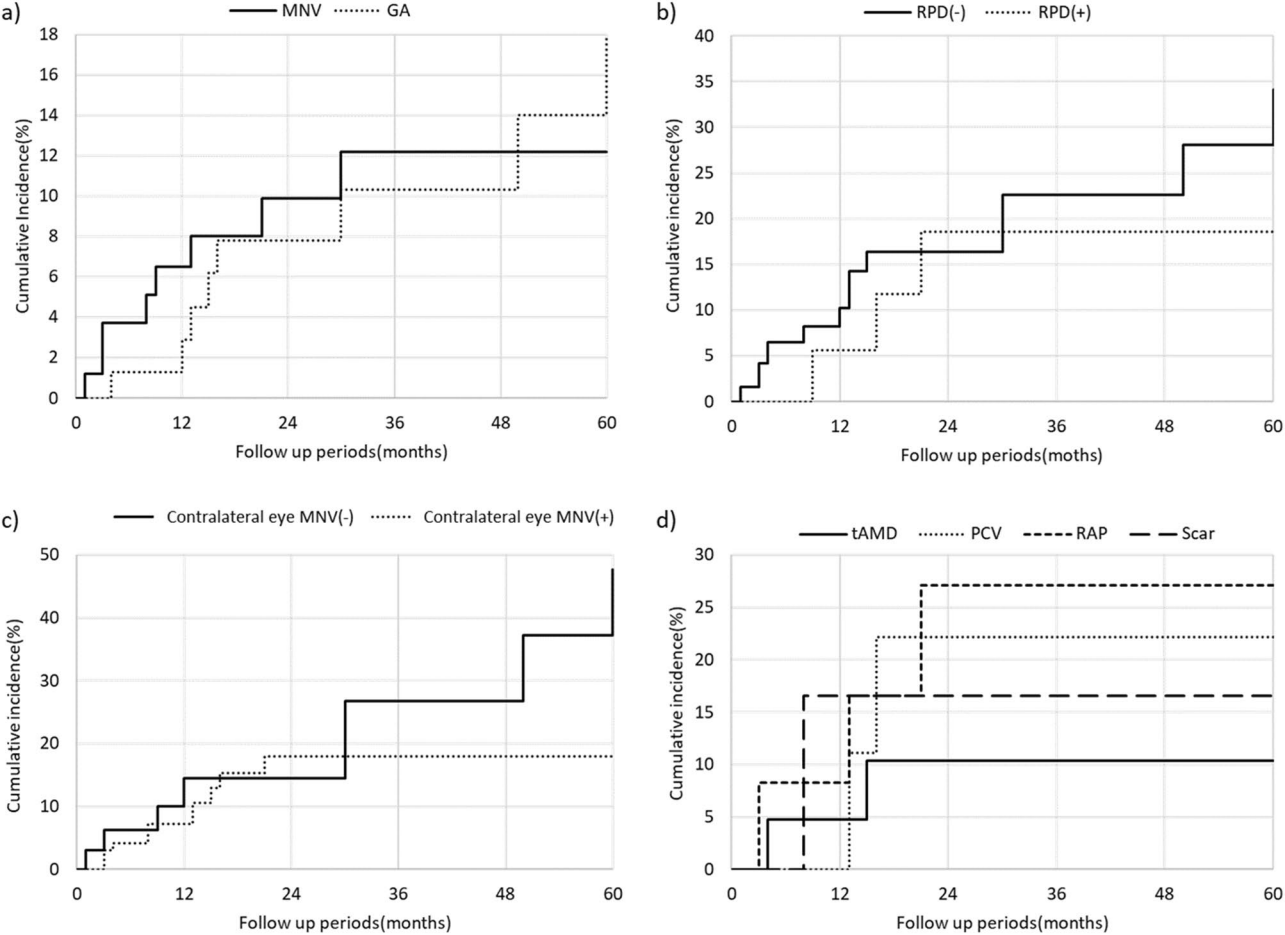
(**a**) Cumulative incidence of geographic atrophy (GA) and macular neovascularization (MNV) at the 5-year follow-up. The cumulative incidence of GA was 2.9%, 7.8%, 10.3%, 10.3%, and 17.9% in the 1-year, 2-year, 3-year, 4-year, and 5-year follow-up respectively. The cumulative incidence of MNV was 6.5%, 9.9%, 12.2%, 12.2%, and 12.2% in the 1-year, 2-year, 3-year, 4-year, and 5-year follow-up respectively. (**b**) Cumulative incidence of advanced age-related macular degeneration (AMD) depends on the presence or absence of reticular pseudodrusen (RPD) during a 5-year follow-up. The cumulative incidence of advanced AMD in RPD (+) patients was 5.6%, 18.6%, 18.6%, 18.6%, and 18.6% at the 1-year, 2-year, 3-year, 4-year, and 5-year follow-up respectively. The cumulative incidence of advanced AMD in RPD (−) was 10.2%, 16.4%, 22.6%, 22.6%, and 34.1% at the 1-year, 2-year, 3-year, 4-year, and 5-year follow-up respectively. There was no difference in the incidence of advanced AMD between patients with and without RPD. (*p* = 0.38, log-rank test). (**c**) Cumulative incidence of advanced AMD was dependent on the presence or absence of MNV in the contralateral eye during a 5-year follow-up. The cumulative incidence of advanced AMD in MNV (+) in the contralateral eyes was 7.2%, 18.0%, 18.0%, 18.0%, and 18.0% at the 1-year, 2-year, 3-year, 4-year, and 5-year follow-up respectively. The cumulative incidence of advanced AMD in MNV (−) in the contralateral eye was 14.5%, 14.5%, 26.7%, 26.7%, and 47.7% at the 1-year, 2-year, 3-year, 4-year, and 5-year follow-up respectively. There was no difference in the incidence of advanced AMD between patients with and without RPD. (*p* = 0.17, log-rank test). (**d**) Cumulative incidence of advanced AMD was dependent on the MNV subtypes in the contralateral eye during a 5-year follow-up. The cumulative incidence of advanced AMD in the contralateral eye with retinal angiomatous proliferation, polypoidal choroidal vasculopathy, typical neovascular AMD, and scarring was 27.1%, 22.2%, 10.4%, and 16.6% in the 5-year follow-up, respectively.

**Figure 2:**
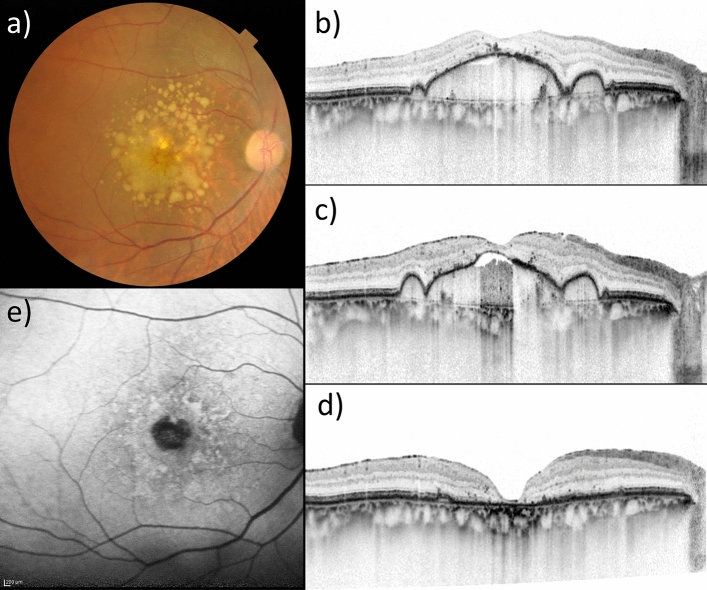
Drusenoid PED in the right eye of a 71-year-male. (**a**) Baseline color fundus photograph of the right eye. (**b**) Baseline horizontal OCT scan showed that the drusenoid PED width and height were 3943 µm and 307 µm, respectively. (**c**) RPE defect and choroidal hypertransmission was seen 3 months before the GA development. (**d**) PED collapse was seen 18 months after the initial presentation. (**e**). (**f**) Hypoautofluorescence was seen at the region corresponding to GA on fundus autofluorescence.

**Figure 3:**
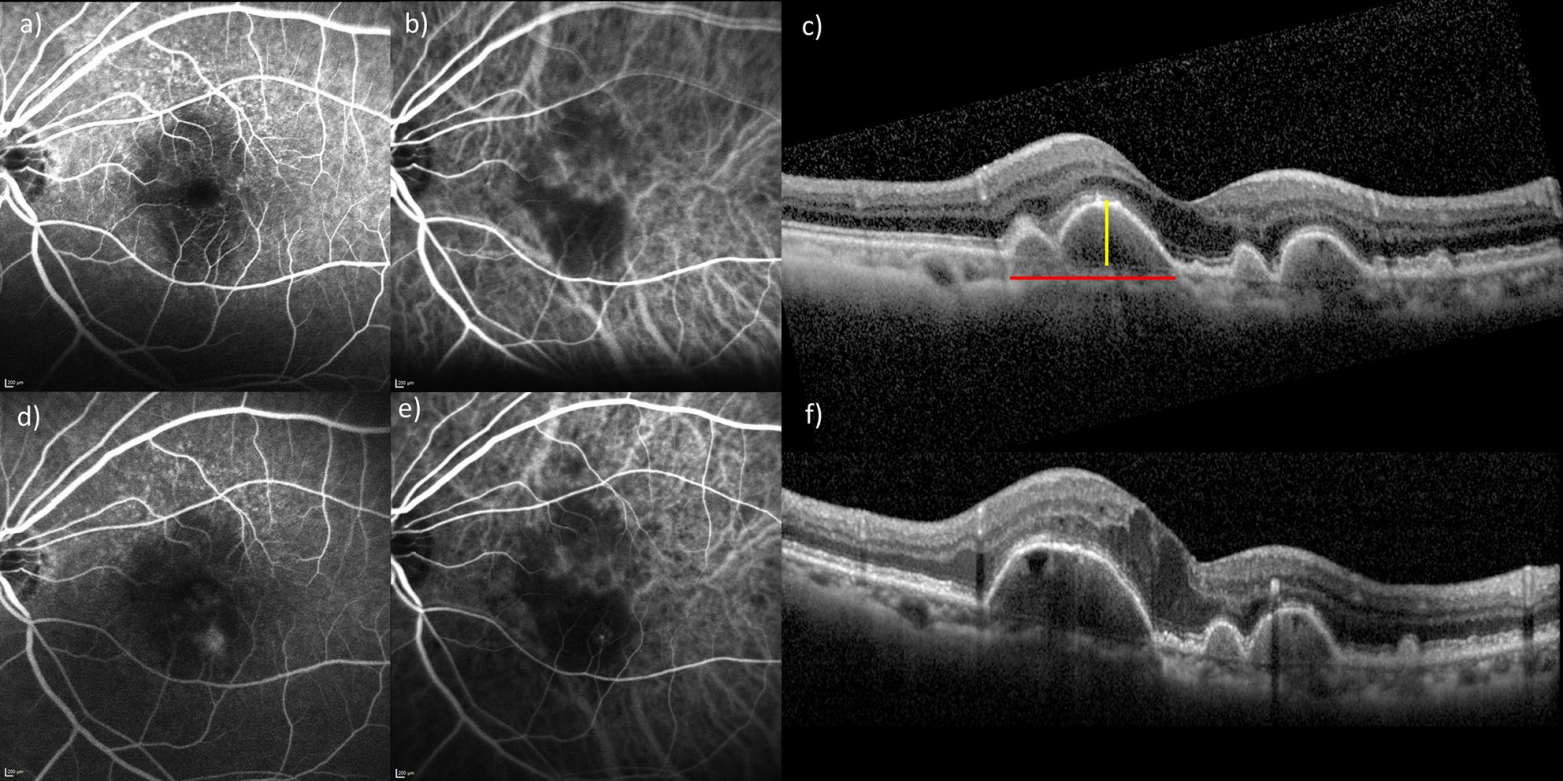
Drusenoid PED in the left eye of an 84-year-female. (**a**) Fluorescein angiography (FA) showed numerous hyperfluorescence in the region superior to the macula. (**b**) Indocyanine green angiography (ICGA) showed no evidence of macular neovascularization. (**c**) Baseline horizontal OCT scan showed that the drusenoid PED width (red line) and height (yellow line) were 877 µm and 154 µm, respectively. (**d**) FA showed a hyperfluorescent spot on the region inferior to the macula. (**e**) On ICGA, a hot spot was observed on the point corresponding to a hyperfluorescent spot on the FA. (**f**) A horizontal OCT scan showed intraretinal fluid 21 months after the initial presentation.

During the study period (mean follow-up period: 33.2 ± 21.6 months), 16 eyes progressed to advanced AMD. MNV and GA developed in eight and eight eyes, respectively. Figure 1a shows the cumulative incidence of GA and MNV at the 5-year follow-up. Representative eyes developing GA and RAP are shown in Figs. 2 and 3, respectively. The cumulative incidence of GA and MNV at the 5-year follow-up was 17.9% and 12.2%, respectively. In eight eyes developing MNV, two eyes and six eyes exhibited typical neovascular and RAP, respectively. In eight eyes developing MNV, the presence of MNV in the contralateral eye was in 5 eyes (62.5%). In eight eyes developing GA, the presence of MNV in the contralateral eye was in 3 eyes (37.5%).

Reticular pseudodrusen was observed in 19 eyes (22.4%). Figure 1b shows the cumulative incidence of advanced AMD depending on the presence or absence of reticular pseudodrusen. There was no significant difference in the development of advanced AMD between eyes with or without reticular pseudodrusen. (*p* = 0.38, log-rank test).

There was no statistical difference in the development of advanced AMD between eyes with and without MNV in the contralateral eye. (Fig. 1c, *p* = 0.17, log-rank test).

**Figure 4:**
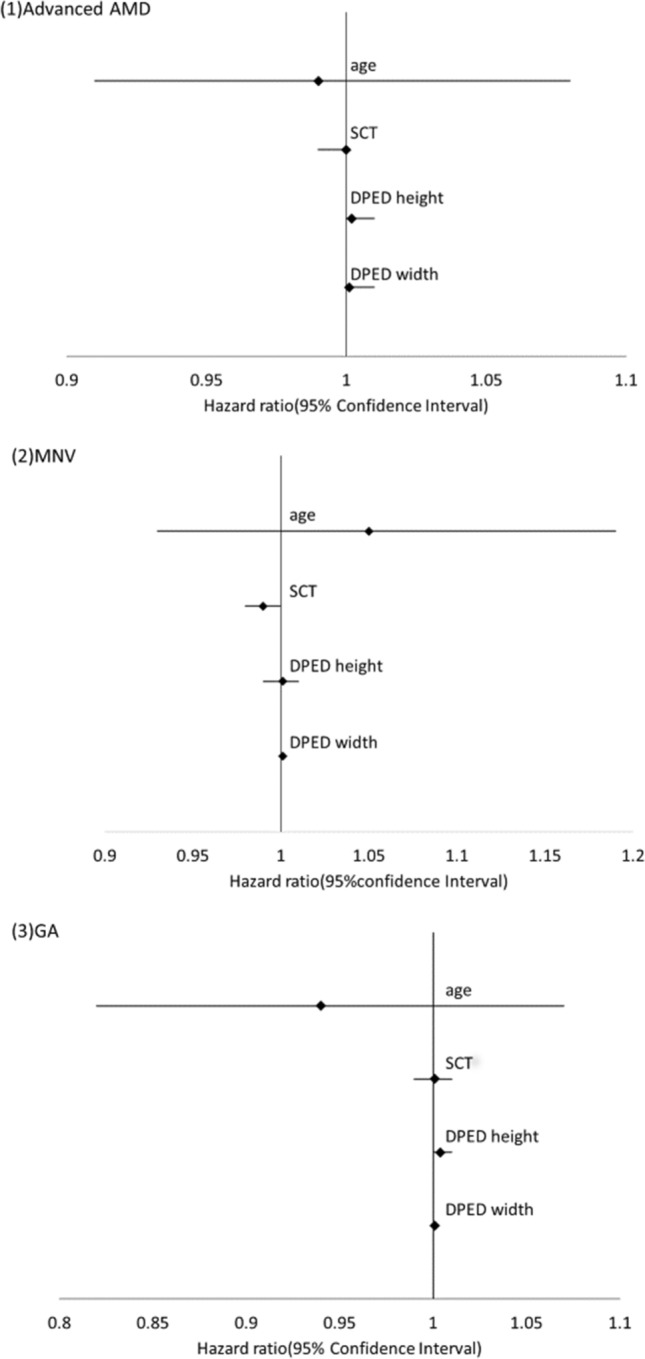
Forest plots showing the hazard ratio associated with advanced AMD, MNV and GA.

To investigate the baseline risk factors for advanced AMD, we employed Cox regression analysis (Table 2). Baseline drusenoid PED width was the only risk factor for progression to advanced AMD (*p* = 0.0026). In addition, we analyzed the baseline risk factor for MNV and GA and found that the baseline drusenoid PED width was associated with MNV (*p* = 0.024), but not with GA (*p* = 0.12). Figure 4 shows the forest plots regarding the risk of advanced AMD, MNV, and GA. In eyes with contralateral eyes involving the MNV, the cumulative incidence of advanced AMD depending on the MNV subtype in the contralateral eye is shown in Fig. 1d. In the MNV subtypes in the contralateral eye, RAP was the highest.

**Table 2:** Cox regression analysis associated with the development of (1) advanced age-related macular degeneration, (2) macular neovascularization, (3) geographic atrophy.

Variables	Β	*p* value	Hazard ratio (95% CI)
**(1)**			
Mean age (years)	− 0.0056	0.90	0.99 (0.91–1.08)
Gender Male	− 0.83	0.15	0.44 (0.14–1.35)
Presence of RPD	− 0.43	0.57	0.65 (0.15–2.89)
SCT (µm)	− 0.005	0.24	1.00 (0.99–1.00)
DPED height (µm)	0.002	0.36	1.002 (1.00–1.01)
DPED width (µm)	0.0008	0.0026	1.001 (1.00–1.001)
Contralateral eye with MNV (Category 4 in AREDS)	− 1.23	0.34	0.29 (0.024–3.63)
Use of anti–VEGF drug in the contralateral eye	1.04	0.40	2.8 (0.25–32.0)
Baseline log MAR VA	1.08	0.57	3.0 (0.07–129.0)

	β	*p* value	Hazard ratio
**(2)**			
Mean age (years)	0.052	0.42	1.05 (0.93–1.19)
Gender Male	− 0.83	0.35	0.44 (0.077–2.48)
Presence of RPD	− 0.13	0.90	0.88 (0.11–6.70)
SCT (µm)	− 0.009	0.19	0.99 (0.98–1.00)
DPED height(µm)	0.001	0.77	1.001 (0.99–1.01)
DPED width(µm)	0.001	0.024	1.001 (1.00–1.001)
Contralateral eye with MNV (Category 4 in AREDS)	− 2.1	0.23	0.12 (0.004–3.87)
Use of anti-VEGF drug in the contralateral eye	1.4	0.39	4.1 (0.17–99.1)
Baseline log MAR VA	2.7	0.31	14.5 (0.086–2419.1)

	β	*p* value	Hazard ratio (95% CI)
**(3)**			
Mean age(years)	− 0.06	0.36	0.94 (0.82–1.07)
Gender Male	− 0.65	0.42	0.53 (0.11–2.52)
Presence of RPD	− 0.96	0.46	0.38 (0.03–4.81)
SCT (µm)	0.0008	0.89	1.001 (0.99–1.01)
DPED height (µm)	0.004	0.21	1.004 (1.00–1.01)
DPED width (µm)	0.001	0.12	1.001 (1.00–1.001)
Contralateral eye with MNV (Category 4 in AREDS)	− 9.31	0.95	–
Use of anti-VEGF drug in the contralateral eye	9.6	0.95	–
Baseline log MAR VA	− 1.0	0.76	0.38 (0.001–179.0)

CI: confidence interval, RPD: reticular pseudodrusen, SCT: subfoveal choroidal thickness, DPED: drusenoid pigment epithelial detachment, MNV: macular neovascularization, log MAR: logarithm minimum angle of resolution, BCVA: best-corrected visual acuity.

Table 3 shows the comparison of baseline characteristics between patients who developed GA and those who developed MNV. There were no differences in baseline factors between the two groups. There were no significant differences in baseline characteristics between patients with MNV and GA.

**Table 3:** Comparison of baseline characteristics between patients developing macular neovascularization (MNV) and geographic atrophy (GA).

	MNV (n = 8)	GA(n = 8)	*P* value
Mean age (years)	80.4 ± 5.2	73.5 ± 7.2	0.083
Gender Male	3 (37.5%)	4 (50.0%)	0.61
Presence of RPD	2 (25.0%)	1 (12.5%)	0.52
SCT (µm)	180 ± 68	232 ± 66	0.16
DPED height (µm)	213 ± 109	246 ± 189	0.96
DPED width (µm)	2509 ± 1087	2124 ± 1605	0.57
Baseline log MAR VA	0.18 ± 0.18	0.02 ± 0.12	0.083

RPD: reticular pseudodrusen, SCT: subfoveal choroidal thickness, log MAR: logarithm minimum angle of resolution, BCVA: best-corrected visual acuity.

## Discussion

This study is the first and the largest study to report the incidence and risk of advanced AMD in eyes with drusenoid PED in Asians. In this study, the 5-year cumulative incidence of advanced AMD was approximately 30%. (MNV: 12.2%, GA:17.9%) In eyes developing MNV, the incidence of RAP was the highest followed by typical neovascular AMD in subtypes of MNV. The baseline PED width was associated only with advanced AMD.

The presence of large drusen and confluent drusen is a well-known risk factor for advanced AMD, including MNV and GA^6,7^. Considering the formation process in drusenoid PED including large drusen aggregation and coalescence, the prevalence of drusenoid PED is considered lower compared with conventional (hard/soft) drusen. A total of 387(8.1%) out of 4757 participants had drusenoid PED in at least one eye^4^. However, there have been no reports regarding the prevalence of drusenoid PED in epidemiological studies except AREDS, and there are a few studies investigating the natural history of drusenoid PED using SD-OCT in clinic-based studies^5,8,9^.

In AREDS, 42% of eyes with drusenoid PED developed advanced AMD within a 5-year follow-up. In AREDS2, the number of risk alleles in *ARMS2* A69S were associated with advanced AMD in eyes with drusenoid PED^10^. Although risk genetic variants were not included as variables in the analysis, approximately 30% of 85 eyes developed advanced AMD in the present study, which is lower compared with Caucasians.

Balaratnasigam et al. conducted a longitudinal study investigating 21 eyes with large drusenoid PED (mean PED width:2427 µm) using SD-OCT and demonstrated that baseline PED volume was associated with PED collapse leading to GA^5^. On the other hand, Fragiotta et al. studied the risk factors for neovascular AMD using 73 eyes with intermediate AMD and revealed that the presence of drusenoid PED increased the risk for neovascular AMD and the PED width, but not height, which was associated with the development of neovascular AMD^8^. The results are consistent with the results of the present study. Assuming that the drusenoid PED is a hemisphere with a radius “R,” PED width and PED height can represent “2R” and “R,” respectively, although PED height is actually less than “R” because the top of the drusenoid PED is usually flattened. This indicates that the PED width is more closely associated with the PED volume than the PED height, and the results between the studies might be almost the same. However, the PED width was associated with MNV (*p* = 0.004), but not with GA (*p* = 0.12) in the present study. This might be because the number of participants was small, or the follow-up period was short.

The mechanism of the MNV development is not fully understood. In the previous study, it histologically revealed that lipid was packed underneath PED^11^. It has been considered that lipid signaling promotes the development of neovascularization in ocular tissues^12^. Given that lipid volume is associated with signal strength, it might be reasonable that PED width is associated with MNV.

Regarding subfoveal choroidal thickness, a recent study reported that subfoveal choroidal thickness decreased in eyes with drusenoid PED just before the PED collapse. However, we could not find any association between baseline subfoveal choroidal thickness and GA development^9^.

Several studies in Asia have studied the 5-year incidence of second eye involvement in contralateral eyes with MNV^13–18^. In eyes with unilateral PCV and unilateral neovascular AMD, the 5-year incidence of second eye MNV involvement was 9.3% and 11.3%, respectively^13^. In pseudodrusen eyes with contralateral eyes involving MNV, the 5-year incidence of advanced AMD was 45%^16^. Considering the previous results, eyes with drusenoid PED in the contralateral eyes with MNV are considered to have a similar risk for MNV to the fellow eye with unilateral neovascular AMD and they have a lower risk for advanced AMD compared with pseudodrusen eyes with contralateral eyes involving MNV. It has been reported that the presence of reticular pseudodrusen increases the risk for GA and MNV in the fellow eye of patients with unilateral MNV compared to patients without this condition^19,20^. However, the presence of reticular pseudodrusen did not increase the risk of advanced AMD in eyes with drusenoid PED in the present study. In drusenoid PED eyes with RPD, PED height and width were smaller (height: 148 ± 51 vs 223 ± 137, *p* = 0.013, width: 1404 ± 1170 vs 1724 ± 1041, *p* = 0.13) compared with those without RPD. This might be why RPD is not a risk of advanced AMD including GA and MNV in the present study. Although further studies are needed, it can be concluded that reticular pseudodrusen might not be associated with an increased risk of advanced AMD in eyes with drusenoid PED.

In the AREDS classification^7^, 51 patients with unilateral MNV and the rest of 34 patients are classified into Category 4 and Category 3, respectively. As shown in Table 2 and Fig. 1C, there was not a significant difference in the development of advanced AMD between the 2 groups. Although further studies with large samples would be needed, the AREDS category was not associated with advanced AMD in the present study.

Of the eight eyes developing MNV, retinal angiomatous proliferation was the most common and it was seen in six eyes. In MNV subtypes, retinal angiomatous proliferation is associated with greater large drusen and thinner choroidal thickness^21^. Given that drusenoid PED is the merge of large drusen, it is reasonable that drusenoid PED is likely to develop retinal angiomatous proliferation.

The baseline characteristics did not differ between patients who developed MNV and GA in the study. This might be because the number of patients in both groups was small. Therefore, a large-scale study is needed accordingly.

The limitations of this study should be mentioned. The major limitation was the retrospective design of the study. The follow-up interval and SD-OCT scan protocol differed between the participants and institutes. Second, our SD-OCT analyses did not include the retinal microstructure analyses. It might be possible to predict the risk of advanced AMD more accurately if detailed retinal microstructure analyses were performed. Third, only 16 eyes developed advanced AMD although it is estimated that the event rate is 30%. A prospective and longer follow-up study is needed to confirm the results of this study.

In summary, the 5-year incidence of advanced AMD was approximately 30% in eyes with drusenoid PED among elderly Japanese patients. Baseline PED width was associated with advanced AMD.

## Methods

The medical charts of patients with drusenoid PED were retrospectively reviewed between January 2012 and September 2021 at the University of Yamanashi Hospital, Nihon University Hospital, and Kobe University Hospital.

The inclusion criterion in this study was follow-up period equal to or more than 1 month. Drusenoid PED was defined as an aggregation of drusen more than 500 µm in width and 100 µm in height on SD-OCT, as previously described^22,23^. If both eyes were eligible, the right eye was included in this study. Drusenoid PED width was defined as the drusenoid PED maximum horizontal length at the fovea through the horizontal scan of SD-OCT, and drusenoid PED height was defined as the distance between the bottom of the RPE and Bruch’s membrane at the fovea through horizontal SD-OCT scans. Subfoveal choroidal thickness was also measured as the length between Bruch’s membrane and the chorioscleral border at the fovea.

### Ethics statement

This study was approved by the institutional review board of each institute including University of Yamanashi, Nihon University School of Medicine, and Kobe University School of Medicine, and it was conducted in accordance with the tenets of the Declaration of Helsinki. The committees waived the requirement for obtaining informed consent, given that this was a retrospective observational study of medical records and was retrospectively registered.

At the initial presentation, all participants underwent a comprehensive ophthalmic examination, including measurement of best-corrected visual acuity (BCVA) using a Landolt chart, intraocular pressure, slit-lamp biomicroscopy with a + 78-diopter lens, color fundus photography, and (SD-OCT examination, near infrared reflectance, and fundus autofluorescence (Spectralis, Heidelberg Engineering, Dossenheim, Germany, HRA + OCT). If exudation including subretinal and/or intraretinal fluid was seen in the contralateral eye, fluorescein angiography (FA) and indocyanine green angiography (ICGA) were also performed.

All patients were followed up every 1–3 months at the physicians’ discretion. At follow-up, a comprehensive examination as per the initial examination was performed for all patients. If an exudative change was observed on OCT, FA/ICGA was performed to confirm the MNV subtype. The presence of reticular pseudodrusen was defined as the presence of characteristic imaging by at least one imaging modality, as previously described^24^. Because this study is a multi-institutional study, the case discrimination and measurement of the retinal and choroidal structure were performed by 3 retinal specialists (Y.S, K.T, A.M) in each institute, not setting a reading center.

Statistical analyses were performed using SPSS software (IBM, Tokyo, Japan). Best-corrected visual acuity measured on a decimal scale on a Landolt chart was converted into logarithm minimal angle of resolution (logMAR) for statistical analyses. A chi-squared test was used to compare the categorical variables between the two groups. The Mann–Whitney U test was used to compare the continuous variables between the two groups. A Cox regression analysis was performed to investigate the risk factors for GA, MNV, and advanced AMD. Statistical significance was defined as a *p* value less than 0.05.

Assuming that α is 0.05, the event ratio is 30% and the statistical power is 80% in the Cox regression analysis, the required sample size was 59, confirmed by XLSTAT2020 free available software.

## Data Availability

All data generated or analyzed during this study are included in this article. Further enquiries can be directed to the corresponding author.
